# A new approach for the synthesis of bisindoles through AgOTf as catalyst

**DOI:** 10.3762/bjoc.10.228

**Published:** 2014-09-17

**Authors:** Jorge Beltrá, M Concepción Gimeno, Raquel P Herrera

**Affiliations:** 1 Departamento de Química Inorgánica. Instituto de Síntesis Química y Catálisis Homogénea (ISQCH), CSIC-Universidad de Zaragoza. E-50009 Zaragoza, Spain; 2 Departamento de Química Orgánica. Instituto de Síntesis Química y Catálisis Homogénea (ISQCH), CSIC-Universidad de Zaragoza. E-50009 Zaragoza, Spain

**Keywords:** Ag(I), aldehydes, bisindole, catalysis, indole

## Abstract

A novel approach for the catalyzed formation of bisindolylmethane derivatives (BIMs) is described. This methodology is the unique example where AgOTf has been successfully used for the activation of aldehydes, giving easy access to a broad range of bisindolyl derivatives with excellent results. Moreover, the simplicity and easy operational methodology using a small amount of commercially available AgOTf (1–3 mol %), one of the lowest catalytic charge used in this process to date, makes this procedure an alternative approach for this interesting and appealing reaction.

## Introduction

**Figure 1:**
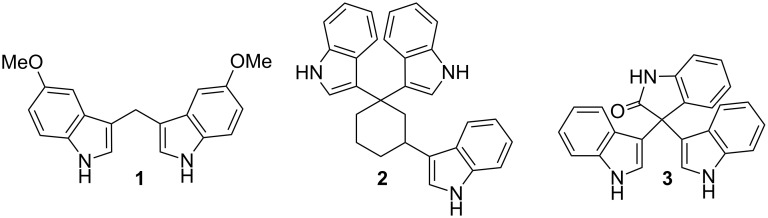
Bisindolyl based important targets: **1** [21], **2** [22] and **3** [23].

Indole is an interesting structural motif present in more than 3000 isolated natural products and embedded in many biological systems [1–3]. Although this field has attracted a considerable attention over the past decades, the development of new synthetic [4–5] and catalytic [6–11] methods leading to functionalized indole derivatives is still an active field because their application in drug discovery [12–14]. Indole is the structural core unit of bisindolylmethane derivatives (BIMs), many of them isolated from marine natural sources [15–16] and are an important class of indole derivatives which exhibit an appealing range of biological properties such as antibacterial, antifungal, antimicrobial or anti-inflammatory, among others (Figure 1) [17]. Interestingly, BIMs have also been found to be potent anticancer derivatives, also having antimetastatic activity, and clinical studies have demonstrated that they could be potentially used as chemotherapeutic agents against numerous forms of cancer [18–20].

The great importance of bisindolylmethanes, has led many efforts dedicated to the development of new strategies for their synthesis after the pioneering work reported by Fischer in 1886 [24–25], and more particularly when aldehydes are involved in their synthesis [26]. Considering the broad scope of application of BIMs together with their interesting biological properties, the development of new efficient, general and eco-friendly protocols using benign catalytic systems with structurally different aldehydes and easy accessible catalysts still remains a continuous demand.

Among the variety of Lewis acids employed in this reaction such as In(OTf)_3_ (5 mol %)/[omim][PF_6_^−^] [27], LiClO_4_ (10 equiv) [28], Cp_2_ZrCl_2_ (5 mol %) [29], Yb-amberlyst (16 mol %) [30], SmI_2_(THF)_2_ (10 mol %) [31], Sb_2_(SO_4_)_3_ (5 mol %) [32], Dy(OTf)_3_ (5–10 mol %) [33], Ln(OTf)_3_ (0.10 M) [34], FeCl_3_·6H_2_O (5 mol %)/[omim][PF_6_^−^] [35], V(HSO_4_)_3_ (10 mol %) [36], Bi(NO_3_)_3_ (2–5 mol %) [37], or CeCl_3_·7H_2_O (10 M) [38], Ag is a good alternative since it is one of the most abundant, cheaper, readily available metals and also displays low toxicity. We disclose here a pioneering strategy for the synthesis of bisindolylmethane derivatives via Ag activation of aldehydes. To the best of our knowledge, this is the first example where catalytic amounts of AgOTf afford the desired bisindolyl products [39–40]. Our approach could significantly broaden the scope of this reaction, providing a new and convenient protocol and avoiding some reported drawbacks, such as the use of toxic metal ions, expensive solvents, long reaction times, high temperature, anhydrous conditions, tedious work-up, low product yields, higher catalyst loading, reaction limited to aromatic aldehydes and formation of large amounts of wastes or byproducts.

## Results and Discussion

**Scheme 1:**
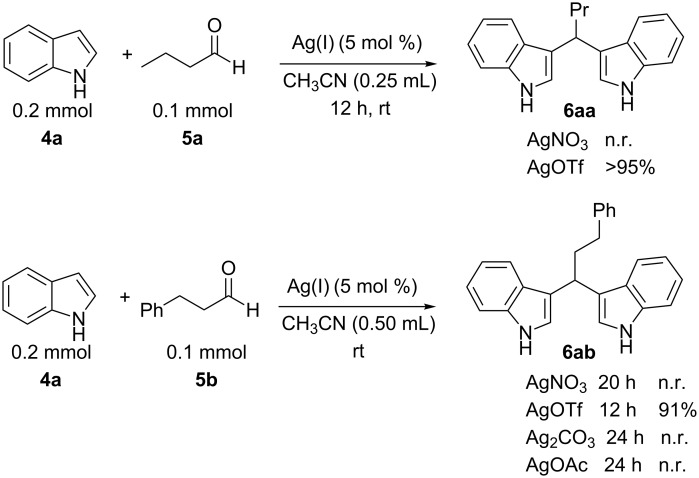
Test reaction using diverse Ag(I) species.

In the context of our research program focused on the synthesis of new indole derivatives, we centered our attention in the preparation of bisindolylmethane derivatives following a very easy and straightforward procedure, in order to contribute to the development of this field. Accordingly, we decided to evaluate the possibility of preparing BIMs from aldehydes and simple Ag(I) species (Scheme 1).

In order to test the viability of this idea, the investigation was started by exploring the efficiency of four easily accessible Ag(I) species in two reaction models (Scheme 1), and following the course of the process by TLC until no qualitative advance of the reaction was observed. To our delight, AgOTf allowed an almost full conversion in a very clean reaction after less than 12 hours, measuring the conversion of the process by ^1^H NMR. In contrast, AgNO_3_, Ag_2_CO_3_ and AgOAc showed a lack of reactivity for both aldehydes **5a** and **5b**. This could be in agreement with the higher coordination capacity of the tested counterions, while OTf is considered to be low-coordinating with some cations, thus favouring the Lewis acid character of Ag(I) [41–42]. Bearing this promising result in mind, the ensuing screening was performed using AgOTf as catalyst and exploring different key parameters of the reaction in order to reach the best reaction conditions (Table 1).

**Table 1:** Screening of the catalytic synthesis of BIM derivatives **6aa**.^a^

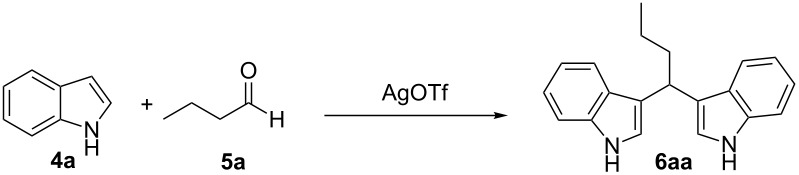				

Entry	Solvent (mL)	AgOTf (mol %)	Time (h)	Conv. (%)^b^

1	Toluene (0.5)	5	3.5	84
2	CH_3_CN (0.5)	5	3.5	84
3	THF (0.5)	5	3.5	89
4	CHCl_3_ (0.5)	5	3.5	93
5	EtOAc (0.5)	5	3.5	78
6	MeOH (0.5)	5	3.5	<5
7	CHCl_3_ (0.5)	3	5	64
8	CHCl_3_ (0.5)	3	18	83
9^c^	CHCl_3_ (0.5)	3	18	88
10^d^	CHCl_3_ (0.5)	3	18	83
11^d^	CHCl_3_ (0.25)	3	18	79
12^d^	CHCl_3_ (0.5)	1	24	89
13^d^	CHCl_3_ (0.25)	1	24	86
14^c,d^	CHCl_3_ (0.5)	1	24	81
15	CHCl_3_ (0.5)	–	24	<5

^a^Experimental conditions: AgOTf (1 or 3 mol %), indole (**4a**, 1 mmol) in 0.25 or 0.5 mL of CHCl_3_ and aldehyde **5a** (0.5 mmol). ^b^Conversion calculated by ^1^H NMR using dimethyl fumarate as internal standard. ^c^Reaction performed using 1.5 equiv of indole. ^d^Reaction performed for 0.5 mmol scale of aldehyde.

In the first screening of solvent, encouraging results were achieved with CHCl_3_ for 5 mol % of catalyst (Table 1, entry 4) in short reactions times (5 h), without heating the reaction. Good results were also obtained for the rest of solvents tested, with the exception of MeOH which exhibited almost no reactivity (Table 1, entry 6). At this point, and because there is a big concern about sustainable chemistry, the next step was centered in decreasing the catalyst loading without reducing the yield of the process, although longer reaction time was required. Interestingly, it was possible to decrease the catalytic amount of the catalyst until 1 mol %, to the best of our knowledge one of the smallest catalyst loading used in this reaction to date (Table 1, entry 12), although longer reaction times were necessary compared with the use of 3 mol % (Table 1, entry 8). In all cases, very clean crude products were found at the end of the process. At this point, the amount of aldehyde was scaled up to 0.5 mmol in order to facilitate the weighing of the catalyst without decreasing the yield of the process (Table 1, entries 10–14). Surprisingly, variation in the dilution of the reaction afforded similar yield for the same reaction time (compare Table 1, entries 10 and 11 and entries 12 and 13). On the other hand, the addition of an excess of indole did not give rise much better results (Table 1, entries 9 and 14). In order to prove the efficiency of this catalytic system the background of the process was tested, demonstrating a lack of reactivity in absence of the catalyst (Table 1, entry 15). With all these results in mind, the scope of the reaction was continued with 1 or 3 mol % of catalyst in CHCl_3_ (0.25–0.50 mL) (Table 2) as the best reaction conditions.

**Table 2:** Scope of the catalytic synthesis of BIM derivatives **6**.^a^

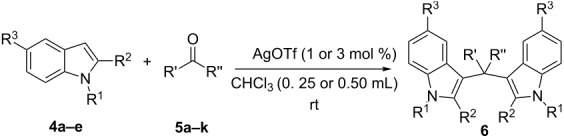							

Entry	Indole	Aldehyde	Solvent (mL)	Cat. (mol %)	Time (h)	Product	Yield (%)^b^

1^c^	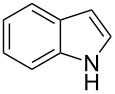 **4a**	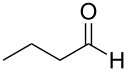 **5a**	5	1	18	**6aa**	>95
2	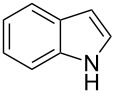 **4a**	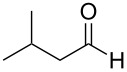 **5b**	0.25	1	18	**6ab**	89
3^d^	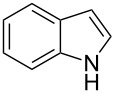 **4a**	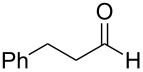 **5c**	0.50	1	15	**6ac**	90
4^d^	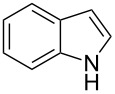 **4a**	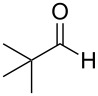 **5d**	0.50	5	48	**6ad**	89
5	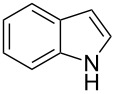 **4a**	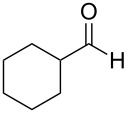 **5e**	0.25	3	18	**6ae**	81
6^d^	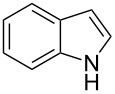 **4a**	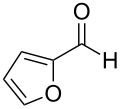 **5f**	0.50	1	16	**6af**	>95
7^d^	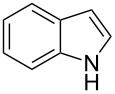 **4a**	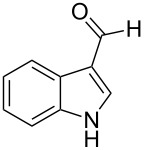 **5g**	0.50	3	24	**6ag**	41
8^d^	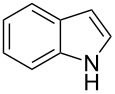 **4a**	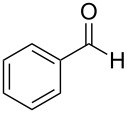 **5h**	0.50	1	18	**6ah**	90
9	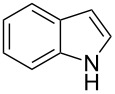 **4a**	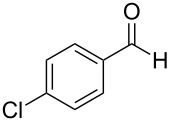 **5i**	0.50	3	18	**6ai**	95
10	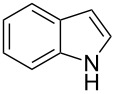 **4a**	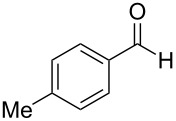 **5j**	0.50	3	24	**6aj**	90
11	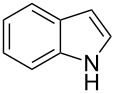 **4a**	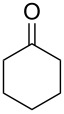 **5k**	0.50	5	24	**6ak**	70
12	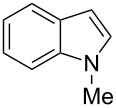 **4b**	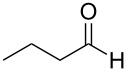 **5a**	0.50	1	18	**6ba**	85
13	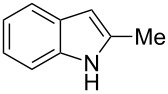 **4c**	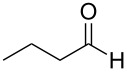 **5a**	0.50	1	18	**6ca**	95
14^d^	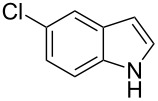 **4d**	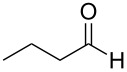 **5a**	0.50	3	24	**6da**	75
15^d^	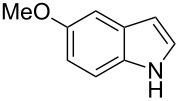 **4e**	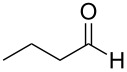 **5a**	0.50	1	8	**6ea**	93

^a^Otherwise indicated: AgOTf (1 or 3 mol %), indole **4a–e** (1 mmol), CHCl_3_ (0.25 mL) and aldehyde/ketone **5a–k** (0.5 mmol). ^b^Isolated yields by flash chromatography (SiO_2_; hexane/EtOAc, 8:2). ^c^Reaction performed using 5 mmol of aldehyde **5a** and 10 mmol of indole (**4a**), for 1.4 g scale. ^d^For 0.2 mmol of aldehyde.

The evaluation of the scope of this Ag(I)-catalyzed methodology was performed testing diverse commercially available aldehydes and indoles giving easy access to a great number of different substituted bisindoles **6** with very good results (Table 2).

As shown above, high yields were achieved in reasonable reaction time using a representative spectrum of aliphatic and aromatic aldehydes (Table 2, entry 1–10). Aldehyde **5d** reacted slowly maybe due to steric factors (3 mol % of catalyst affords 34% yield after 24 h). However, in order to increase the reaction rate preventing the evaporation of **5d**, the process was carried out with 5 mol % of catalyst and a higher yield was achieved (Table 2, entry 4). It is remarkable that the resulting product starting from aldehyde **5g** afforded interesting trisindole analogue **6ag** (Table 2, entry 7). Trisindoles are a less explored class of indole derivatives but with potential anticancer properties [26], and their synthesis is also an appealing challenge. Due to the high insolubility of aldehyde **5g** in the reaction medium, the reaction was performed with 5 mol % of catalyst, however moderate results were obtained in this case. Different solvents were also tested in order to improve the yield of final **6ag** but only a 35% yield was obtained in EtOAc and no reaction in THF, after 2 days. The use of different substituted indoles **4b–e** allowed the desired products to be obtained with excellent results (Table 2, entries 12–15). There is a clear dependence between the electronic environment of the indole and the yield of the final product, since less reactivity was exhibited with indole **4d** (Table 2, entry 14). Given the possibility that product **6ca** could decompose in the column and in order to prevent this drawback and the decomposition of other additional products, the reaction crude was filtered through neutral alumina with Et_2_O, and very clean crude product spectrum was obtained. It is also possible that in some cases the yield could depend on the stability of the starting aldehyde since they are used without previous distillation. This methodology was also tested with the less active and explored ketone **5k** and the reaction rendered a high yield, although 5 mol % of catalyst was required in order to decrease the reaction rate (Table 2, entry 11). Additionally, the possibility of scaling up this process was explored to obtain 1.4 g of product **6ab** (Table 2, entry 2), and successfully completed conversion was achieved after 18 h of reaction with a very clean spectrum (see Supporting Information File 1).

**Scheme 2:**
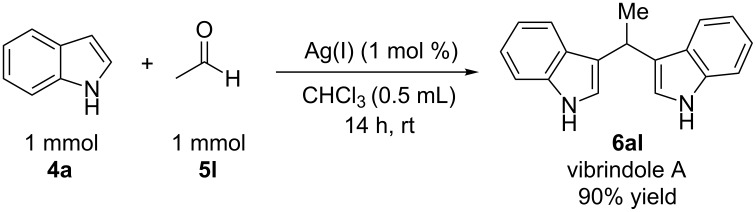
Synthesis of biologically active vibrindole A.

In order to prove the applicability of this methodology, the scope of the reaction was extended to the synthesis of the interesting compound vibrindole A (Scheme 2), an isolated metabolite of the marine bacteria *Vibrio parahaemolyticus*, which is active against *Bacillus subtilis*, *Staphylococcus aureus* and *Staphylococcus albus* as has been previously demonstrated [43].

**Figure 2:**
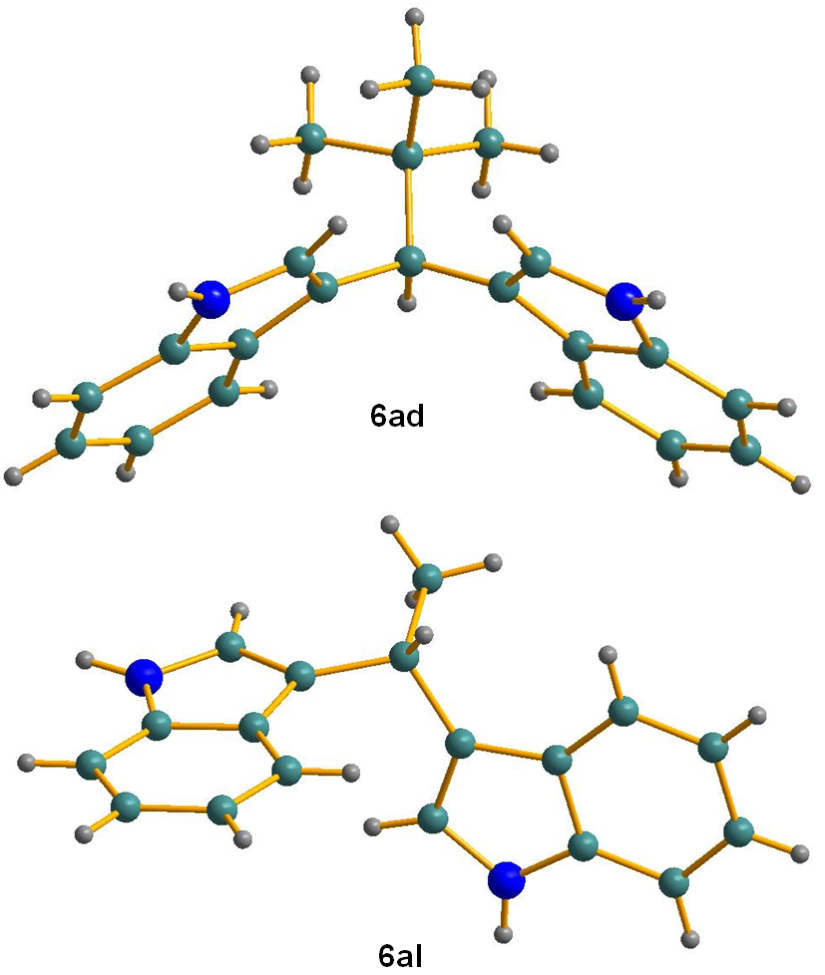
Crystal structures for compounds **6ad** and **6al**.

In order to prevent the loss of acetaldehyde by evaporation (bp 20 °C) and the consequent poorer yield of the final product, instead of 1 equivalent of aldehyde (0.5 mmol) 2 equivalents (1 mmol) were used. The result was 90% of yield compared with 67% for 1 equivalent of acetaldehyde, confirming our concern. It is worth mentioning that this procedure allowed excellent results using 1 mol % of catalyst, one of the lowest catalytic charges used to date. This synthesis represents an easier approach in comparison with previously reported methods for the synthesis of vibrindole A [44–49]. After evaporation of the solvent, a very clean crude product was obtained since acetaldehyde is also evaporated, and the final product was purified by a simple column chromatography. Moreover, we were able to obtain single crystals for compounds **6ad** and **6al** for the first time in the literature (Figure 2) [50].

**Scheme 3:**
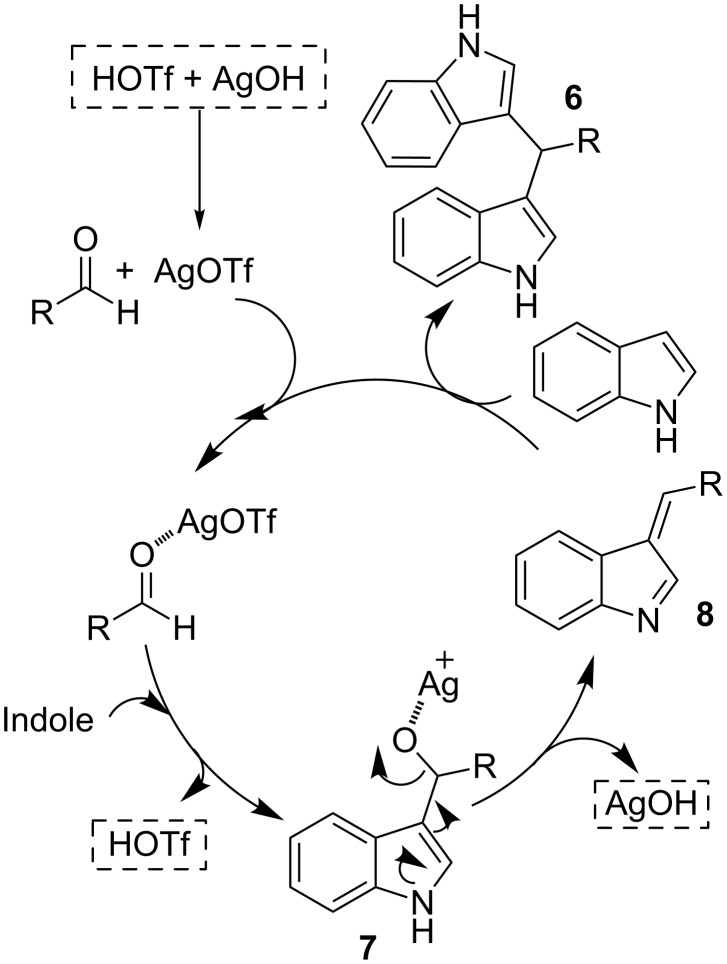
Mechanism of the synthesis of bisindoles through AgOTf catalyst.

Although the mechanism is not clear at this stage, we can assume the same mechanism as that previously proposed for other Lewis acid (Scheme 3). In the course of the reaction after the first addition of one molecule of indole to the aldehyde, unstable intermediate **7** would promote the elimination of Ag(I), in the form of AgOH, to give azafulvene **8** [51–52]. Finally, the iminium **8** would undergo a further addition of a second molecule of indole to produce the final observed products **6** [53].

Since HOTf is released in the second step of the reaction, it is not possible to discard the participation of HOTf in the activation process, since the catalytic ability of these species has been previously invoked by other authors, when metal triflates are used as catalysts in other reactions [54]. Moreover, Brønsted acids have also been employed as promoters of this process [26]. We have performed a comparative study of the reaction between indole (**4a**) and aldehyde **5a**, one with HOTf (1 mol %) and the other with AgOTf (1 mol %), showing that the reaction rate of the process catalyzed by triflic acid is higher (after 6 h the reaction is finished). This is also in agreement with the results observed by Hartwig and co-workers [54]. These findings indicate that AgOTf is the mean catalyst of the process, because although triflic acid could participate in the activation process, it would be consumed in the reaction with the produced AgOH to regenerate AgOTf.

## Conclusion

In summary, we have reported for the first time an unprecedented approach of AgOTf-catalyzed addition of indoles to aldehydes, providing easy access to interesting BIMs with very good yields. This catalytic system has been proven active under mild reaction conditions and effective for a broad application in the synthesis of aliphatic and aromatic substituted bisiindolylmethane derivatives. The small amount of catalyst (1–3 mol %) used in our developed procedure is another advantage being one of the lowest catalytic charge used to date for this process. Furthermore, the simplicity and easy operational methodology makes this procedure an excellent alternative approach for these interesting and appealing reaction products, for which there is still a continuous demand.

## Experimental

Purification of reaction products was carried out by flash chromatography using silica gel (0.063–0.200 mm). Analytical thin-layer chromatography was performed on 0.25 mm silical gel 60-F plates. ^1^H NMR spectra were recorded at 400 MHz; ^13^C APT-NMR spectra were recorded at 100 MHz; CDCl_3_ as the solvent. Chemical shifts were reported in the δ scale relative to the central line of CDCl_3_ (77 ppm) for ^13^C APT-NMR. All commercially available solvents and reagents were used as received. The ^1^H and ^13^C NMR spectra for compounds **6aa** [55], **6ab** [56], **6ac** [57], **6ad** [58], **6ae** [35], **6af** [59], **6ag** [23], **6ah** [60], **6ai** [61], **6aj** [58], **6ak** [57], **6ba** [62], **6ca** [63], **6da** [64], **6ea** [64], are consistent with values previously reported in the literature.

**Representative procedure for the synthesis of bisindoles 6:** To a mixture of catalyst AgOTf (1 or 3 mol %) and indole **4a–e** (1 mmol) in CHCl_3_ (0.25–0.5 mL), aldehyde **5a–k** (0.5 mmol) was further added in a test tube at room temperature. After the reaction time (see Table 2), the crude mixture was filtered through neutral alumina with Et_2_O and the solvent was removed under vacuum. Then, adducts **6** were finally isolated by flash chromatography (SiO_2_; hexane/EtOAc, 8:2). Yield for each final product is reported in Table 2. Final products should be stored under nitrogen atmosphere and in the fridge.

**3,3'-(3-phenylpropane-1,1-diyl)bis(1*H*-indole) (6ac)**


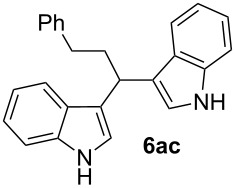


Following the general procedure, compound **6ac** was obtained after 15 h of reaction at room temperature in 90% yield. The ^1^H NMR spectrum is consistent with the values previously reported in [57]. ^13^C APT-NMR (100 MHz, CDCl_3_) δ 142.6 (1C), 136.6 (2C), 128.5 (2C), 128.2 (2C), 127.0 (2C), 125.6 (1C), 121.8 (2C), 121.5 (2C), 120.0 (2C), 119.6 (2C), 119.0 (2C), 111.1 (2C), 37.4 (1C), 34.4 (1C), 33.5 (1C).

**3,3'-(2,2-dimethylpropane-1,1-diyl)bis(1*H*-indole) (6ad)**


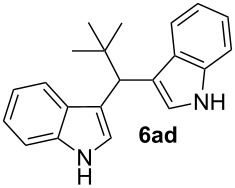


Following the general procedure, compound **6ad** was obtained after 48 h of reaction at room temperature in 89% yield. The ^1^H NMR spectrum is consistent with the values previously reported in [59]. ^13^C APT-NMR (100 MHz, CDCl_3_) δ 135.3 (2C), 128.7 (2C), 121.9 (2C), 121.6 (2C), 119.4 (2C), 119.2 (2C), 119.1 (2C), 110.7 (2C), 43.1 (1C), 35.8 (1C), 29.0 (3C).

## Supporting Information

File 1 ^1^H NMR for all synthesized compounds, ^13^C APT-NMR spectra for compounds **6ac** and **6ad** and crystallographic data for compounds **6ad** and **6al**.
