# Supplementary material for: A new approach for the synthesis of bisindoles through AgOTf as catalyst
Source: Beilstein J Org Chem. 2014 Sep 17;10:2206–14. doi: 10.3762/bjoc.10.228 (PMC4168942; doi:10.3762/bjoc.10.228)
Supplement: File 1 — 1H NMR for all synthesized compounds, 13C APT-NMR spectra for compounds 6ac and 6ad and crystallographic data for compounds 6ad and 6al. [file Beilstein_J_Org_Chem-10-2206-s001.pdf]

Supporting Information

for

A new approach for the synthesis of bisindoles through AgOTf as catalyst

Jorge Beltrá[1], M. Concepción Gimeno*[1] and Raquel P. Herrera*[2]

Address: [1]Departamento de Química Inorgánica. Instituto de Síntesis Química y Catálisis Homogénea (ISQCH), CSIC-Universidad de Zaragoza. E-50009 Zaragoza, Spain and [2]Departamento de Química Orgánica. Instituto de Síntesis Química y Catálisis Homogénea (ISQCH), CSIC-Universidad de Zaragoza. E-50009 Zaragoza, Spain.

E-mail: M. Concepción* Gimeno - gimeno@unizar.es; Raquel P. Herrera* - raquelph@unizar.es

*Corresponding author

^{1}H NMR for all synthesized compounds, ^{13}C APT NMR spectra for compounds 6ac and 6ad and crystallographic data for compounds 6ad and 6al

General experimental methods. Purification of reaction products was carried out by flash chromatography using silical-gel (0.063-0.200 mm). Analytical thin layer chromatography was performed on 0.25 mm silical gel 60-F plates. ^{1}H-NMR spectra were recorded at 400 MHz; ^{13}APT-NMR spectra were recorded at 100 MHz; $CDCl_3$ as the solvent. Chemical shifts were reported in the δ scale relative to central line of $CDCl_3$ (77 ppm) for ^{13}APT-NMR.

Materials. All commercially available solvents and reagents were used as received. The ^{1}H and ^{13}C NMR spectra for compounds **6aa** [1], **6ab** [2], **6ac** [3], **6ad** [4], **6ae** [5], **6af** [6], **6ag** [7], **6ah** [8], **6ai** [9], **6aj** [4], **6ak** [3], **6ba** [10], **6ca** [11], **6da** [12], **6ea** [12], are consistent with values previously reported in the literature.

^{1}H NMR spectrum for the crude of the reaction giving 6aa (400 MHz, $CDCl_3$)

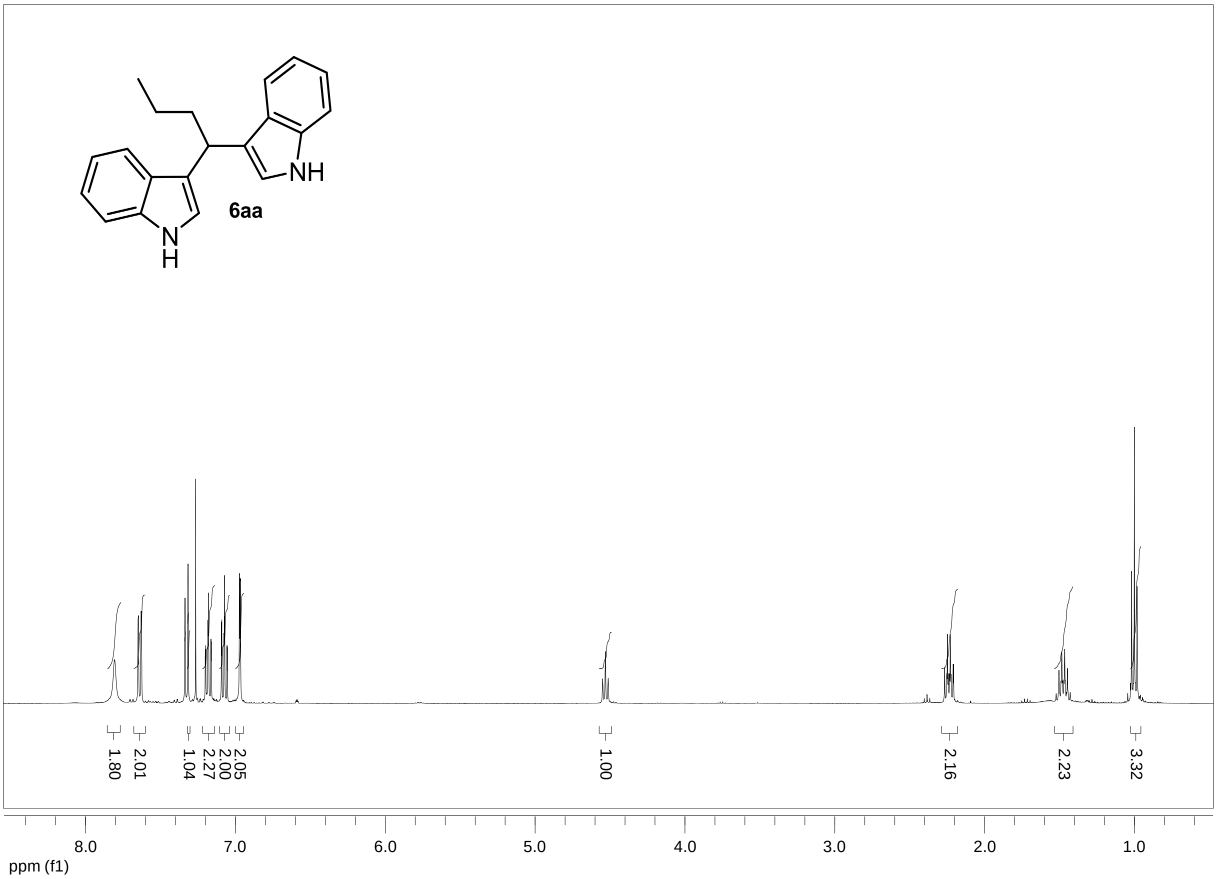

^{1}H NMR spectrum for the crude of the reaction giving 6ab (400 MHz, $CDCl_3$)

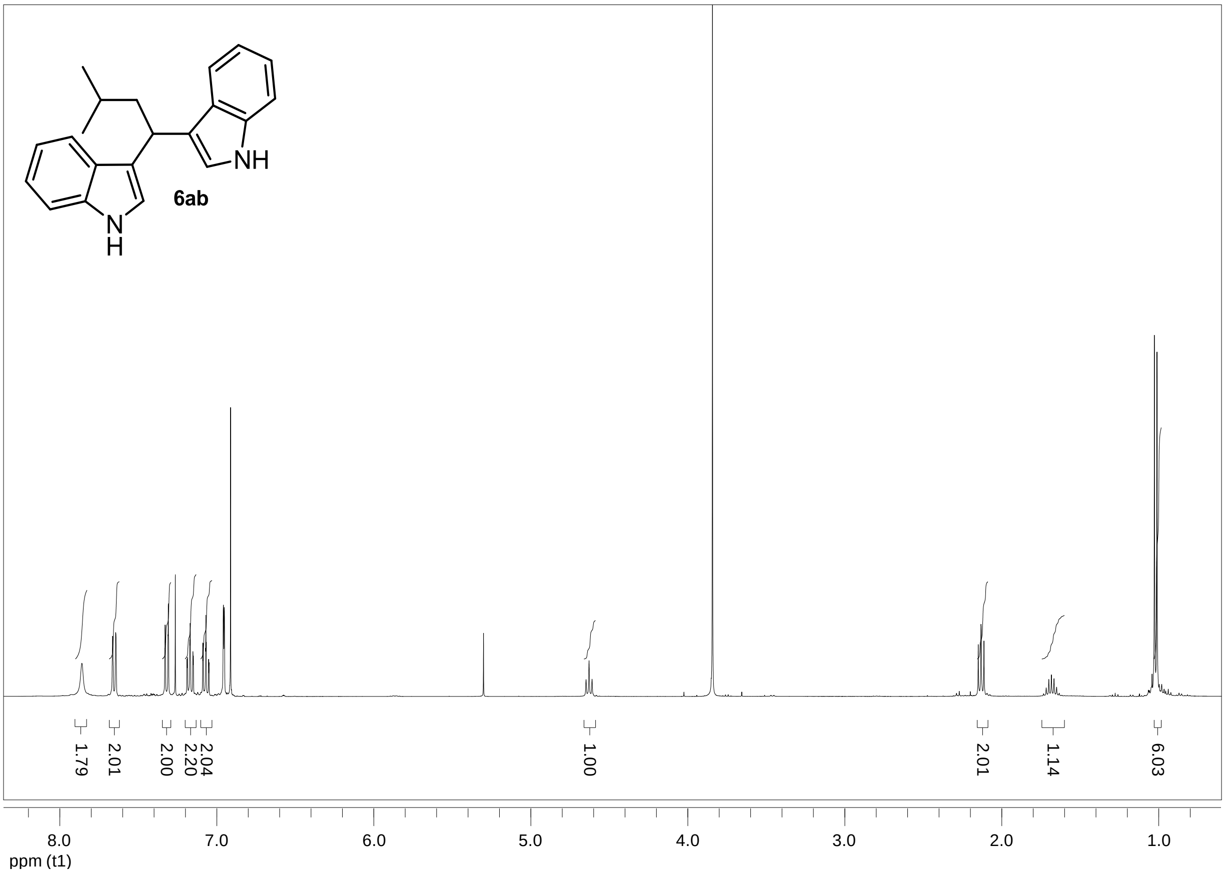

^{1}H NMR spectrum for compound 6ac (400 MHz, $CDCl_3$)

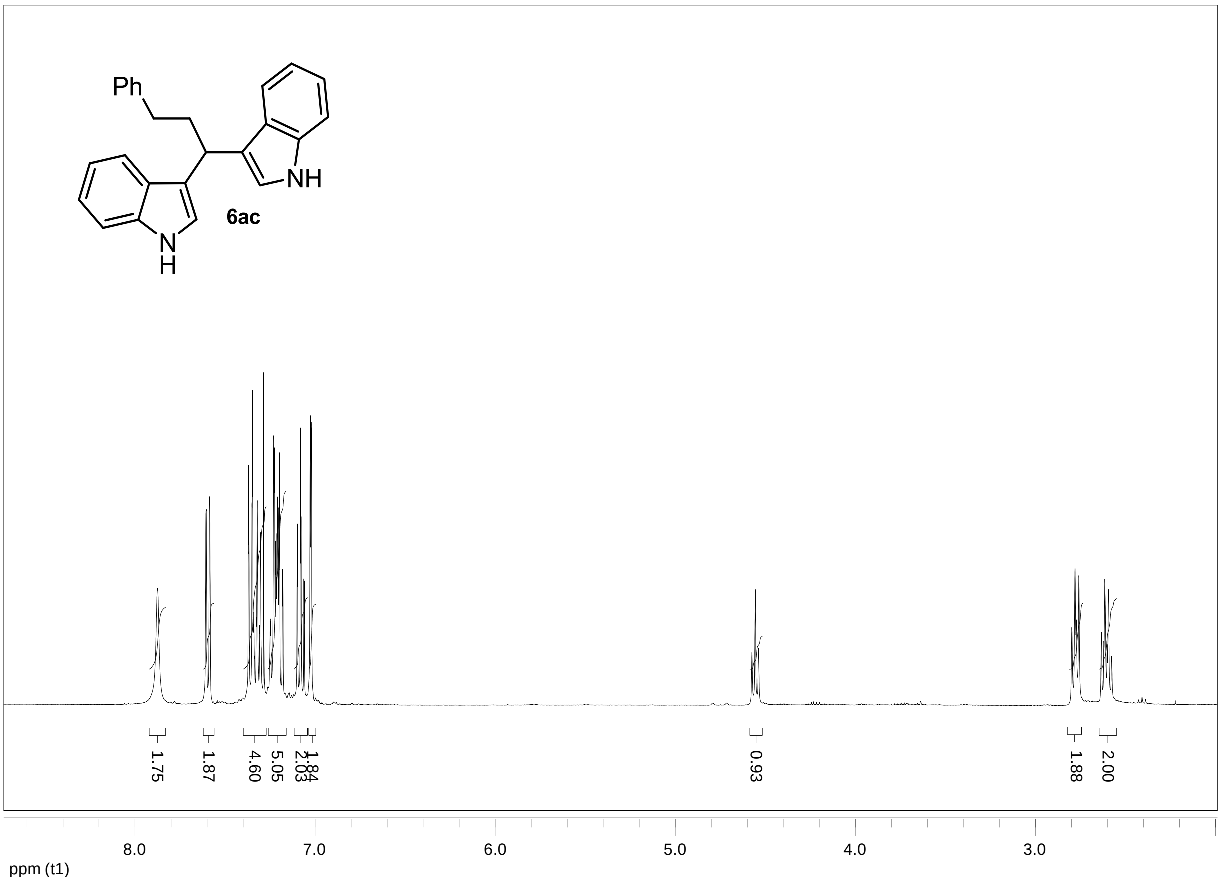

^{13}C NMR spectrum for compound 6ac (100 MHz, $CDCl_3$)

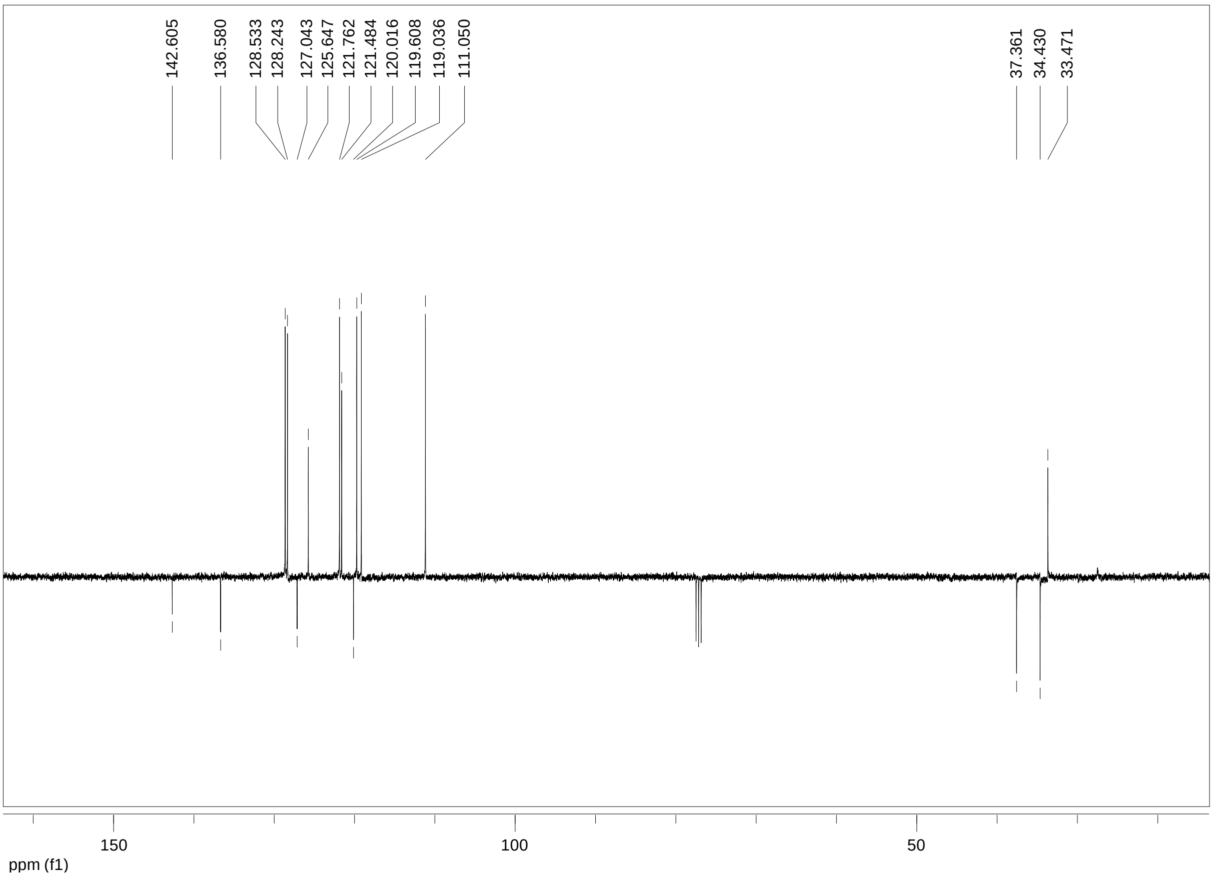

^{1}H NMR spectrum for compound 6ad (400 MHz, $CDCl_3$)

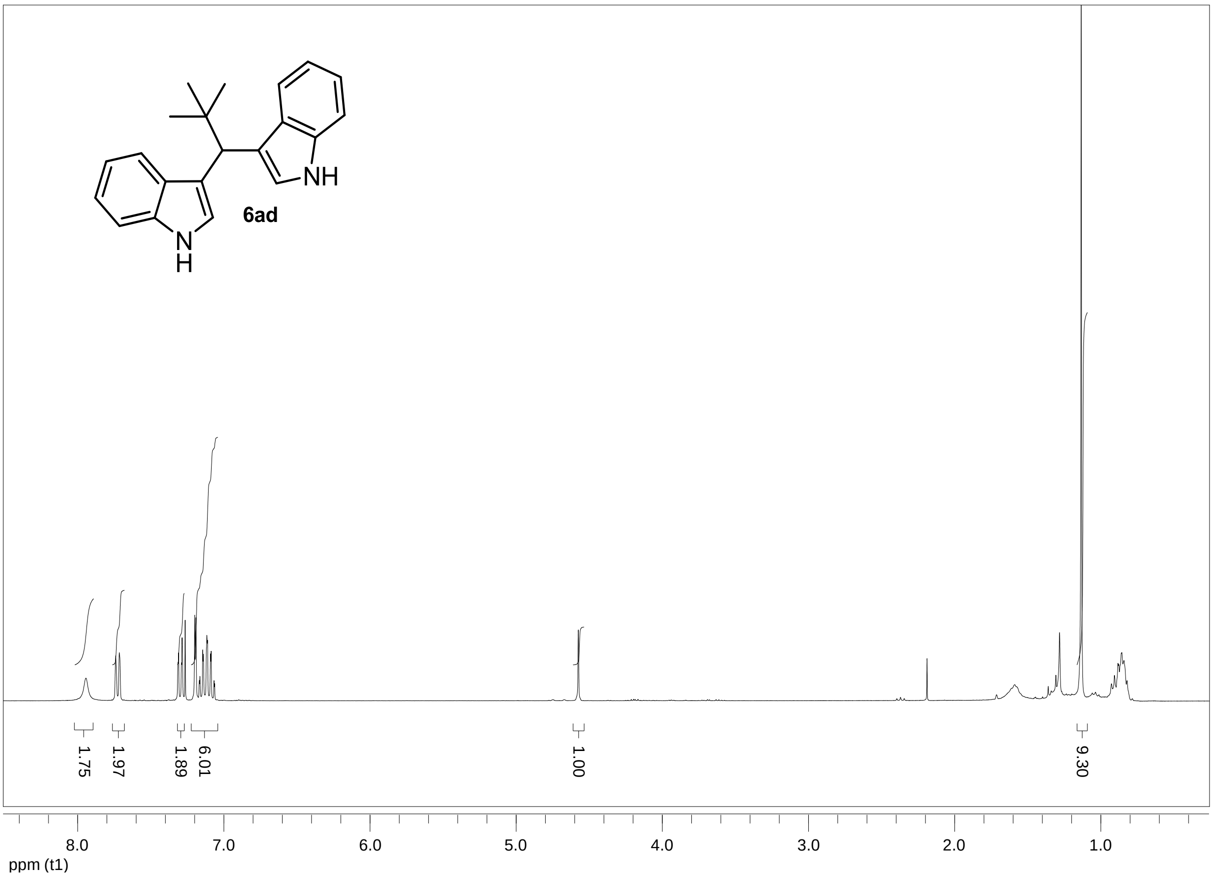

^{13}C NMR spectrum for compound 6ad (100 MHz, $CDCl_3$)

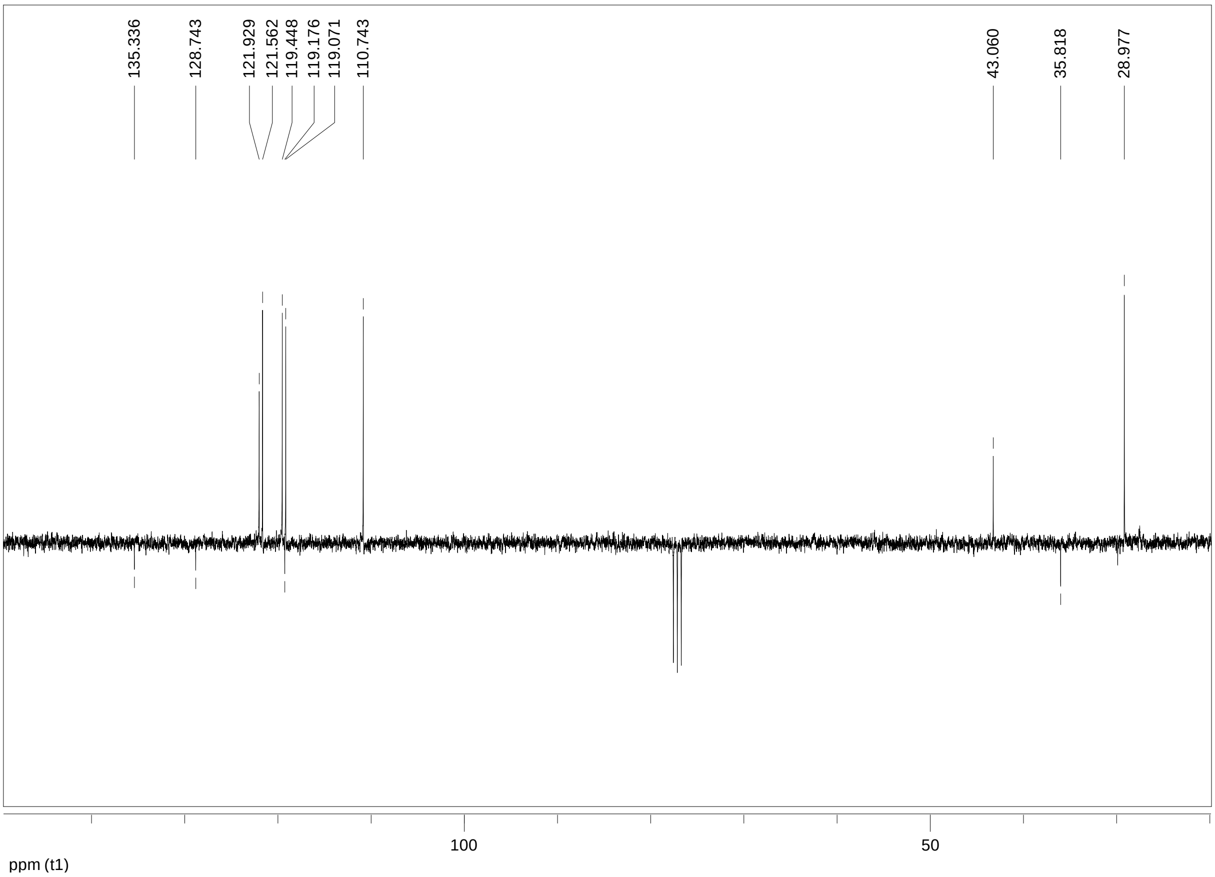

^{1}H NMR spectrum for compound 6ae (400 MHz, $CDCl_3$)

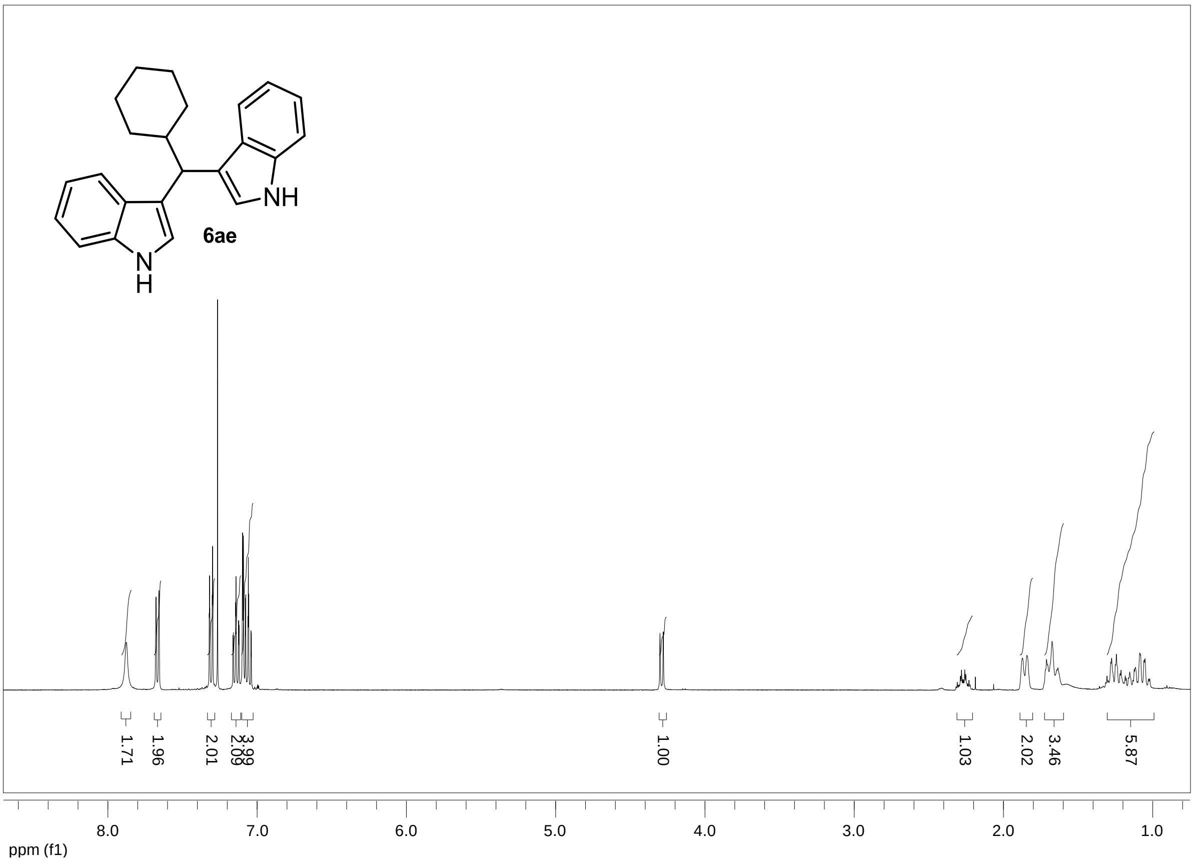

^{1}H NMR spectrum for compound 6af (400 MHz, $CDCl_3$)

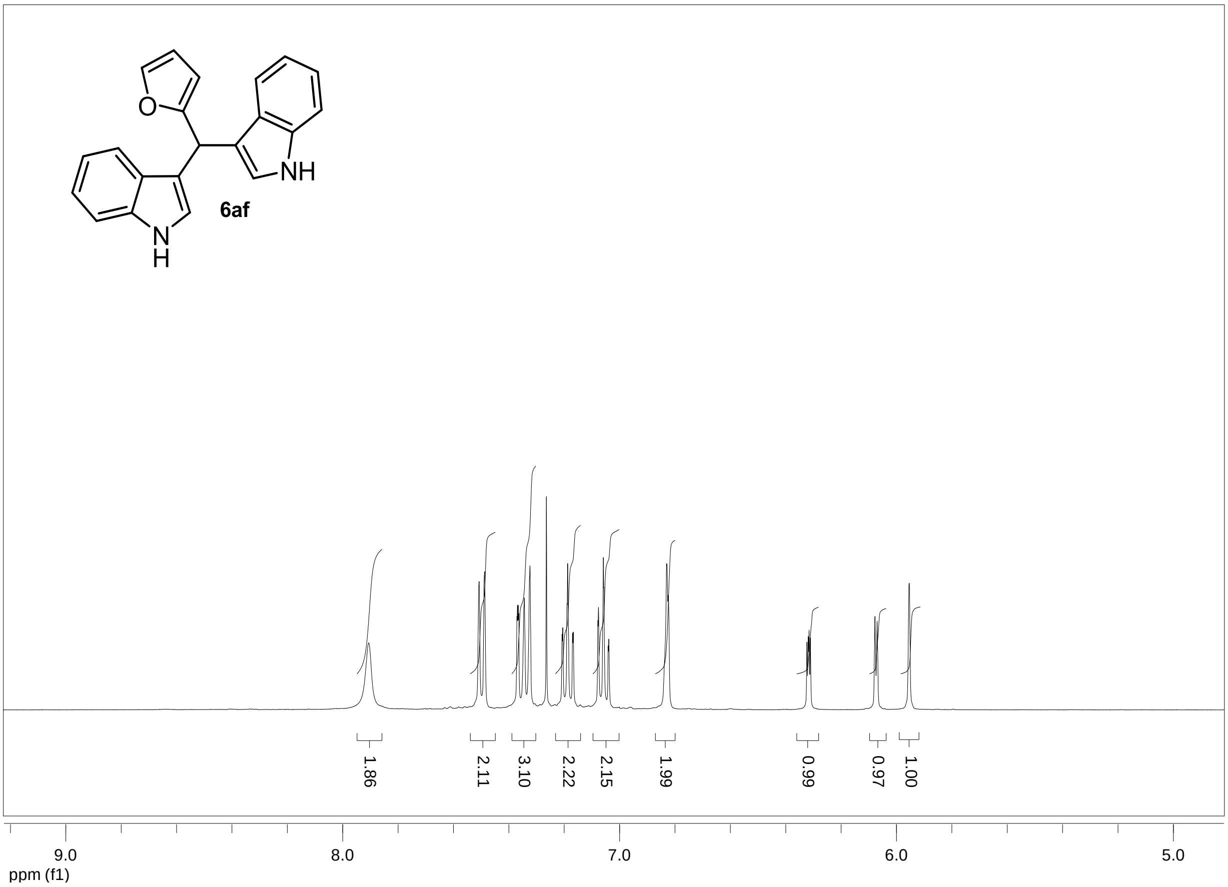

^{1}H NMR spectrum for compound 6ag (400 MHz, $CDCl_3$)

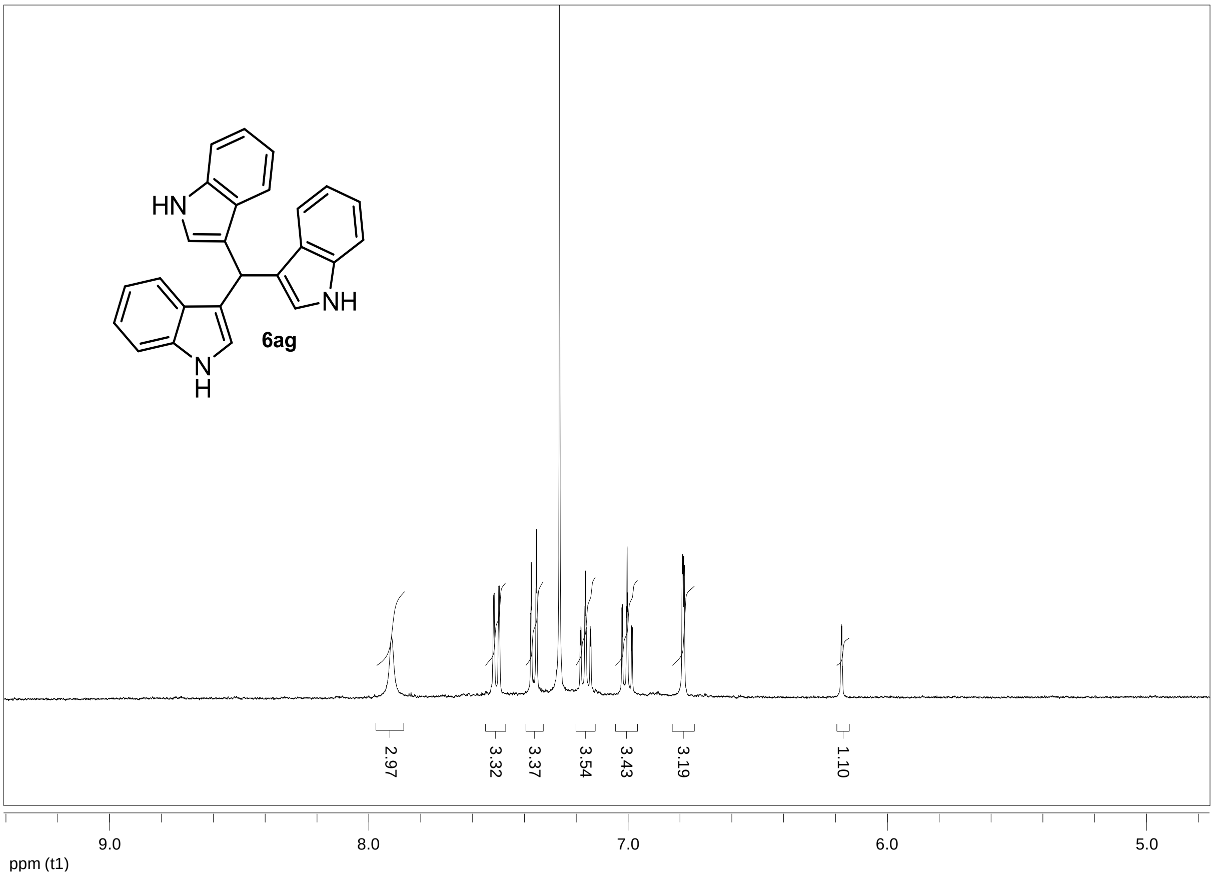

^{1}H NMR spectrum for compound 6ah (400 MHz, $CDCl_3$)

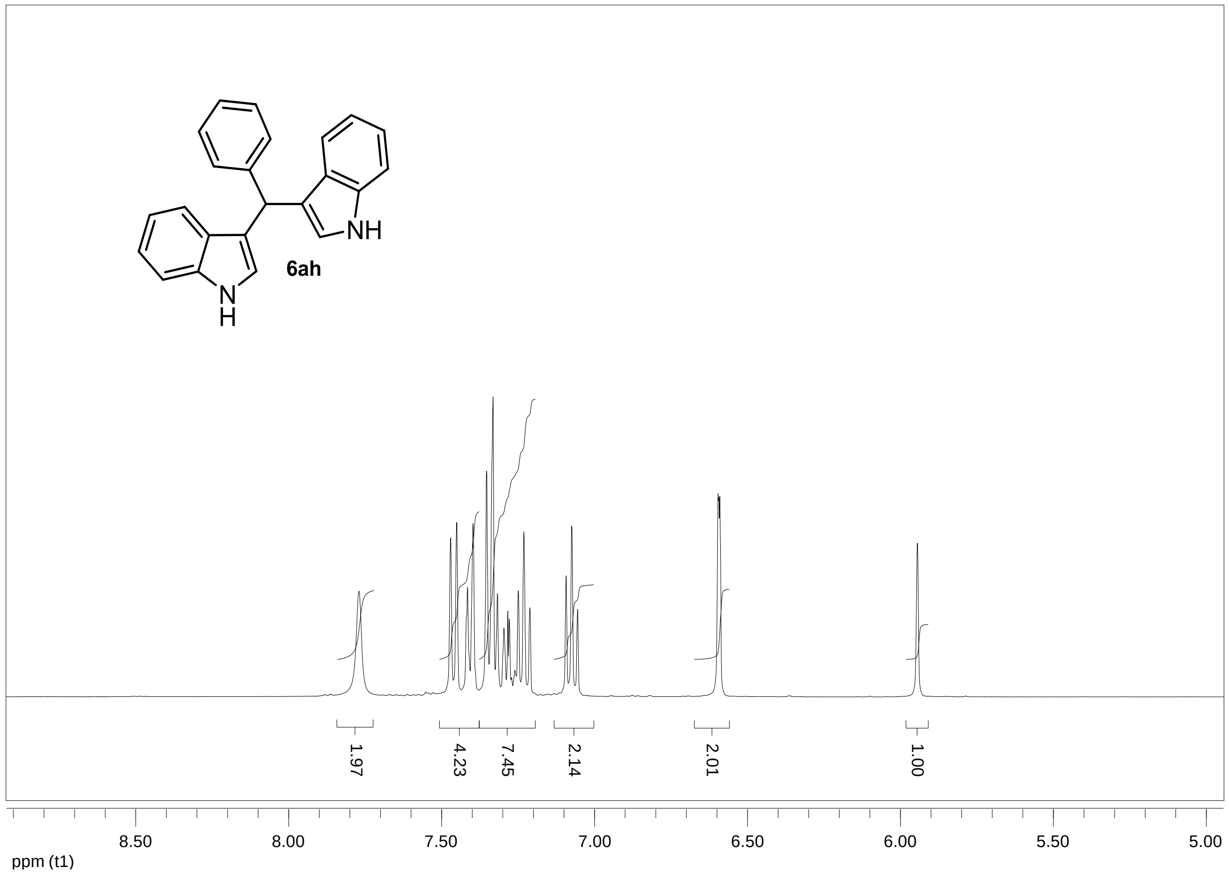

¹H NMR spectrum for the crude of the reaction giving 6ai (400 MHz, $CDCl_3$)

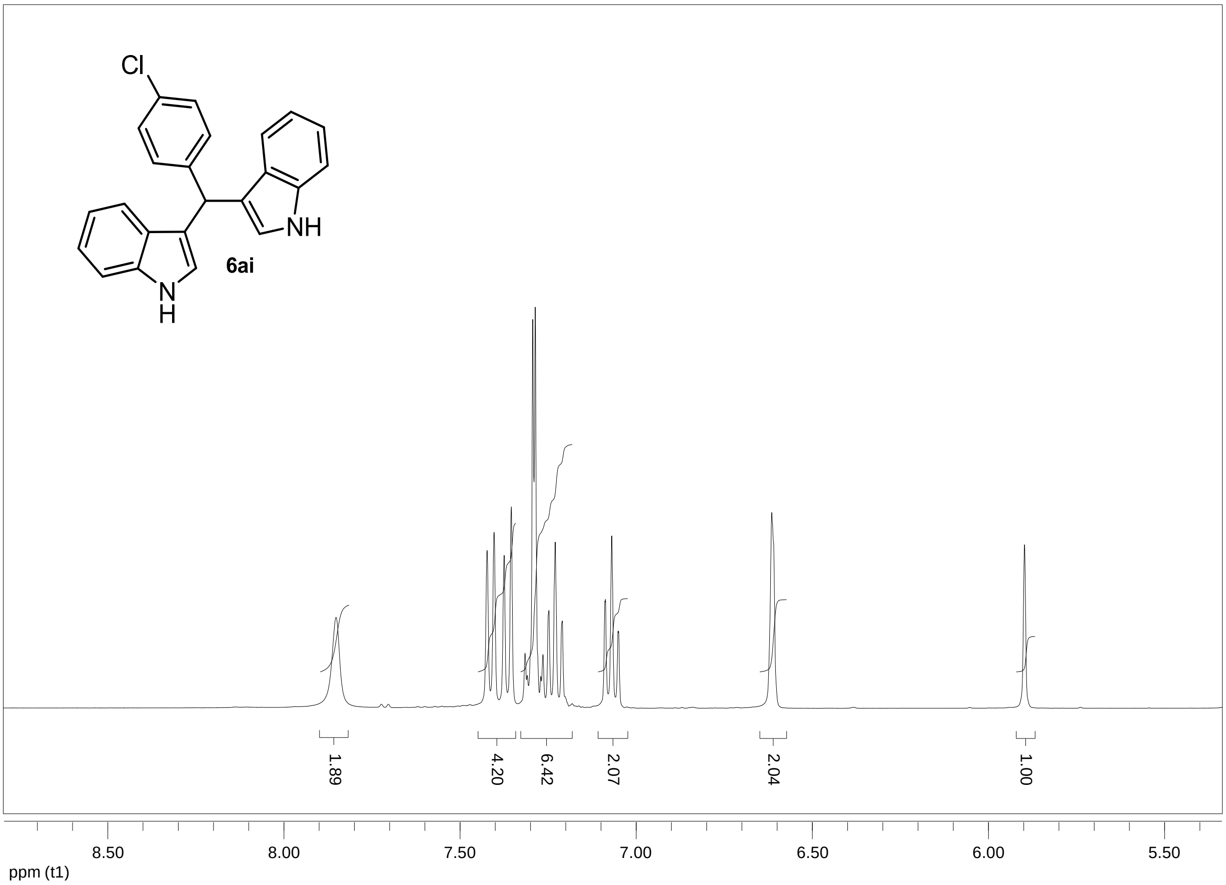

¹H NMR spectrum compound 6aj (400 MHz, $CDCl_3$)

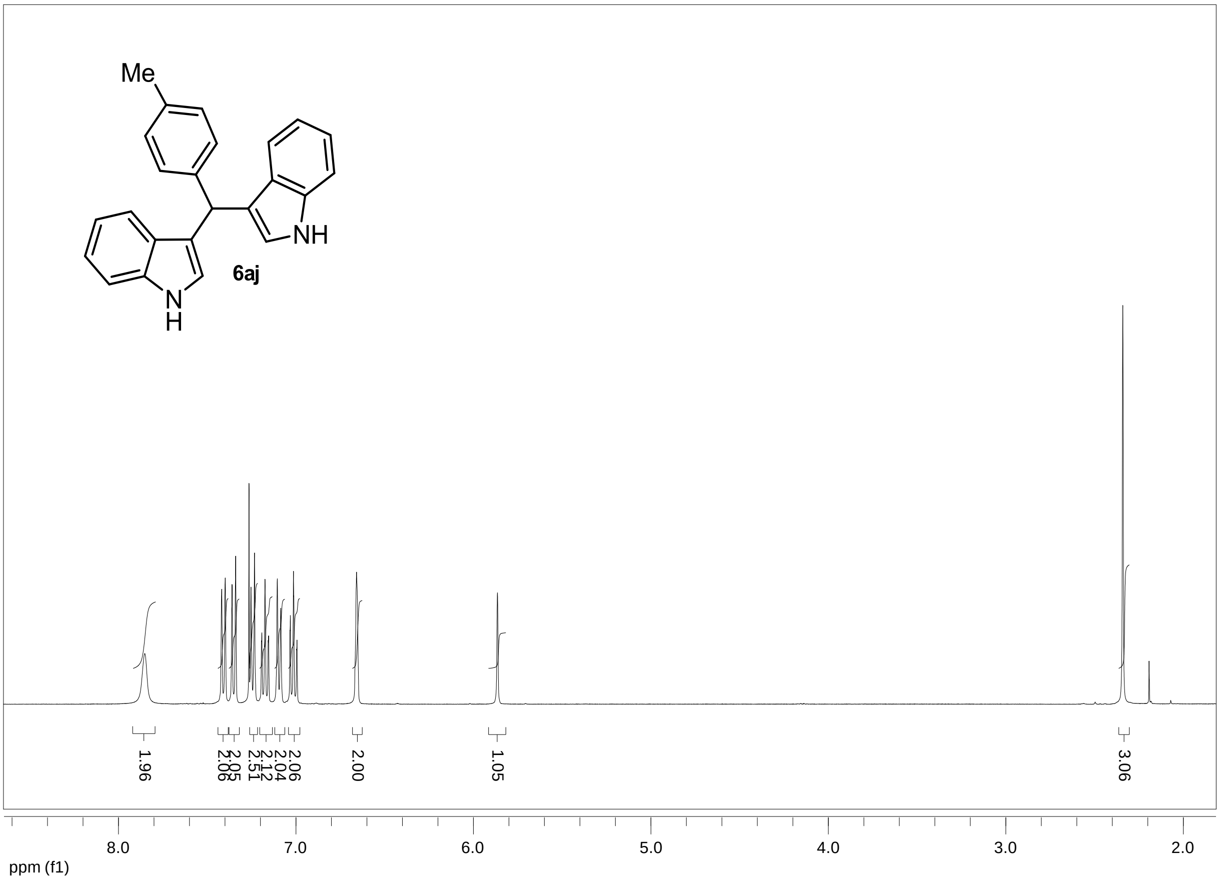

^{1}H NMR spectrum for compound 6ak (400 MHz, $CDCl_3$)

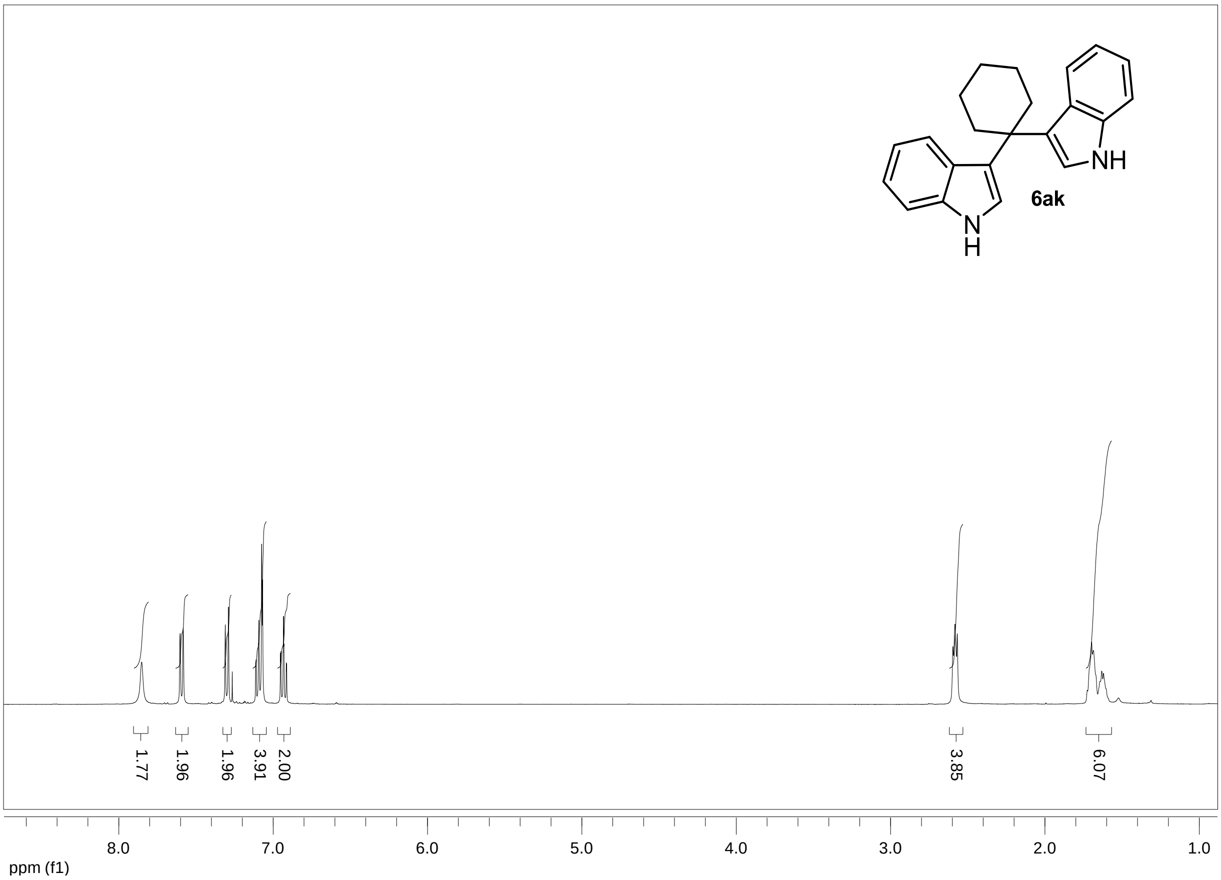

^{1}H NMR spectrum for compound 6ba (400 MHz, $CDCl_3$)

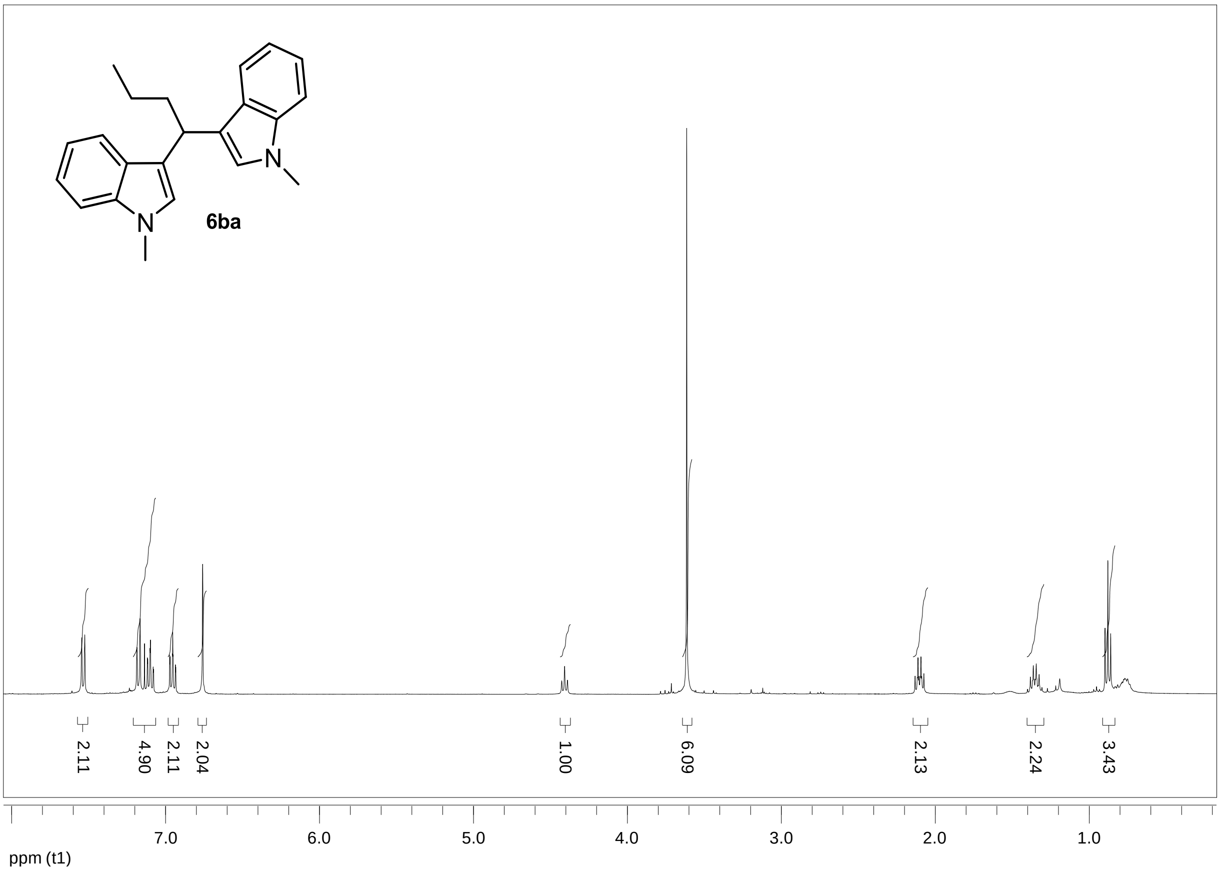

^{1}H NMR spectrum for the crude of the reaction giving 6ca (400 MHz, $CDCl_3$)

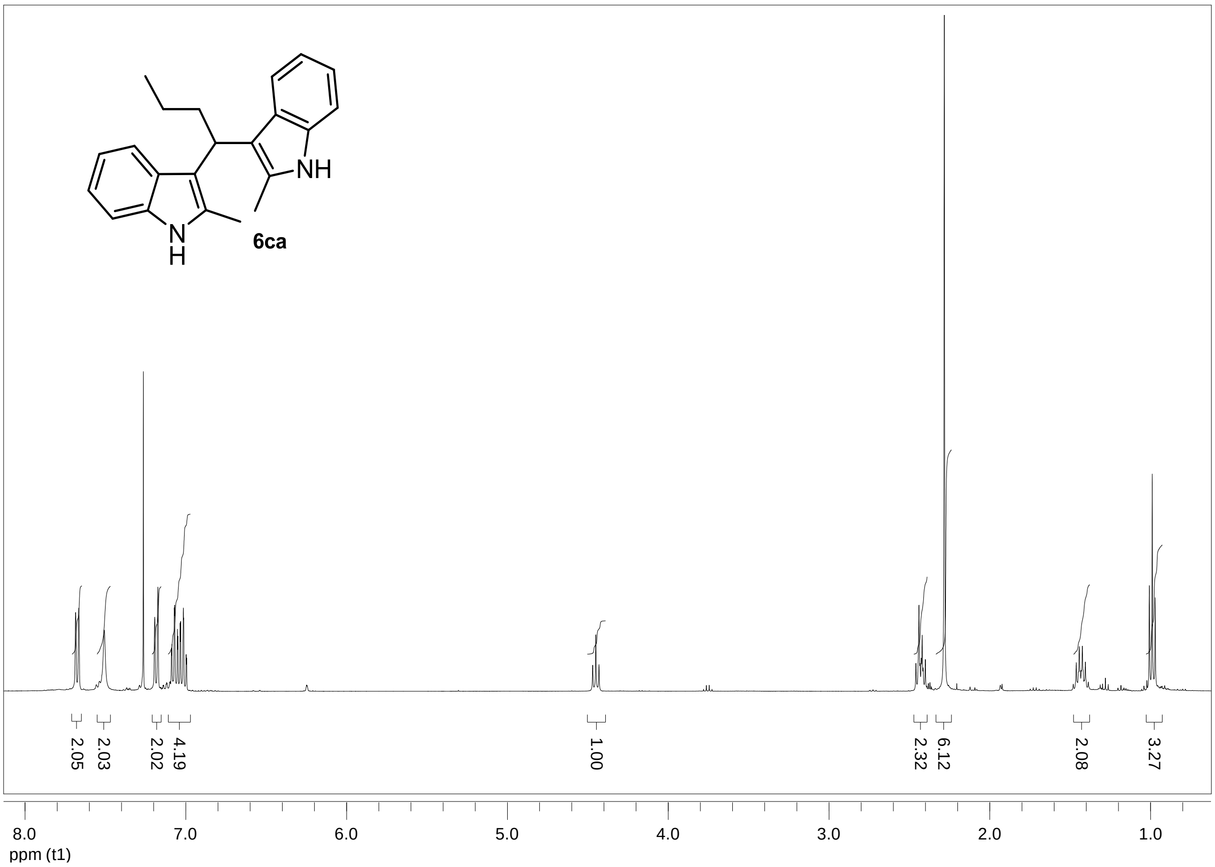

^{1}H NMR spectrum for compound 6da (400 MHz, $CDCl_3$)

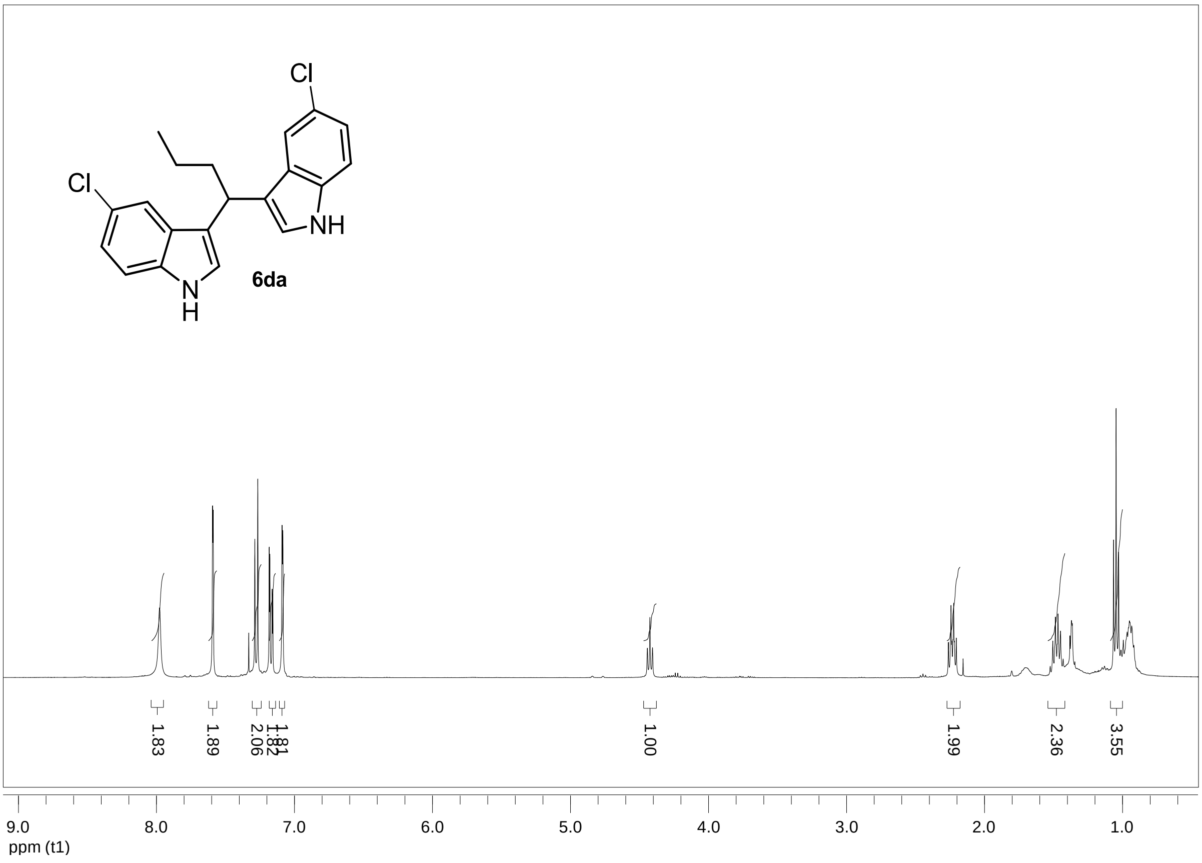

^{1}H NMR spectrum for compound 6ea (400 MHz, $CDCl_3$)

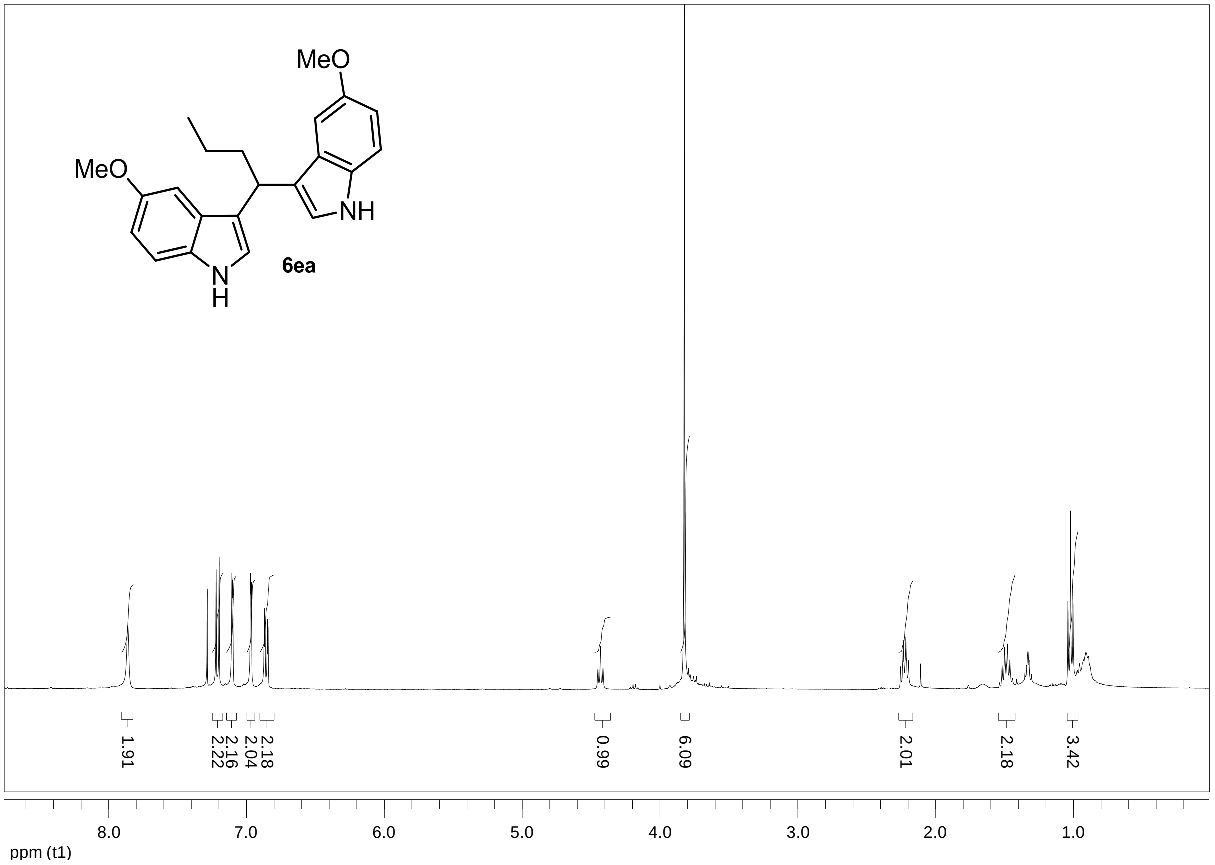

^{1}H NMR spectrum for compound 6al (400 MHz, $CDCl_3$)

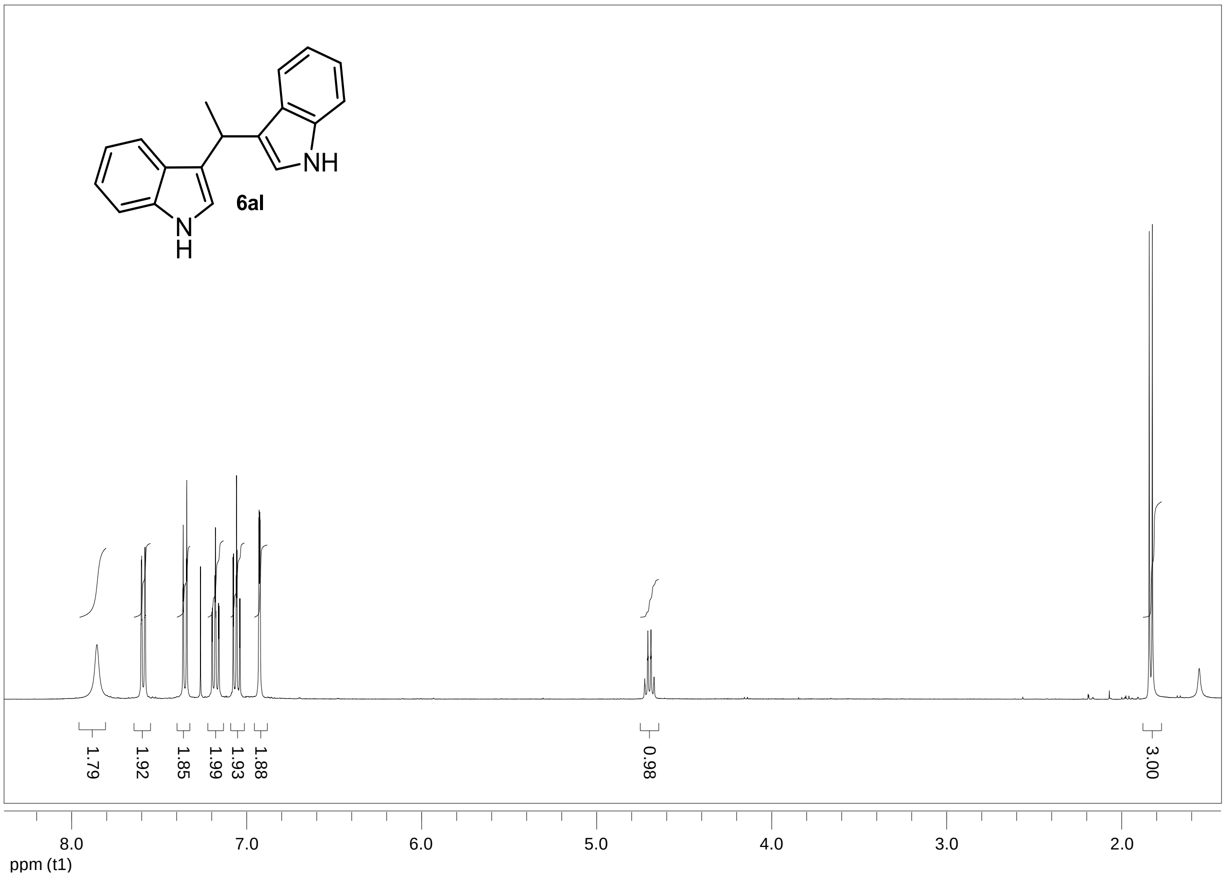

X-RAY CRYSTALLOGRAPHY

The crystals were mounted in inert oil on glass fibers and transferred to the cold gas stream of an Xcalibur Oxford Diffraction diffractometer equipped with a low-temperature attachment. Data were collected using monochromated Mo Kα radiation (λ= 0.71073 Å). Scan type: ω. Absorption correction based on multiple scans was applied using spherical harmonics implemented in SCALE3 ABSPACK scaling algorithm [13]. The structures were solved by direct methods and refined on F^2 using the program SHELXL-97 [14]. All non-hydrogen atoms were refined anisotropically. Hydrogen atoms were included in calculated positions and refined using a riding model, with the exception of NH atoms. Refinements were carried out by full-matrix least-squares on F^2 for all data.

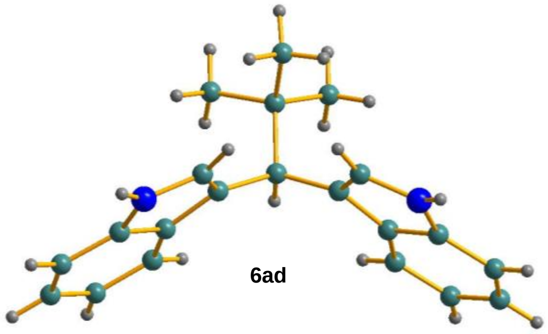

6ad

Crystal data of **6ad**: [$C_{21}H_{22}N_2$], orthorhombic, *Pna2₁*, a = 11.099(2) Å, b = 19.895(4) Å, c = 7.6414(15) Å, Z = 4, M_r = 302.41 g mol^{-1}, V = 1687.3(6) Å^3, D_{calcd} = 1.190 g cm^{-3}, λ(Mo Kα) = 0.71073 Å, T = 100 K, μ = 0.070 mm^{-1}, 9103 reflections collected, 3055 unique (R_{int} = 0.0565), $R1(F_o)$ = 0.0502 [$I > 2\sigma(I)$], $wR2$ (F_o^2) = 0.0852 (all data), GOF = 1.012. CCDC 1007024.

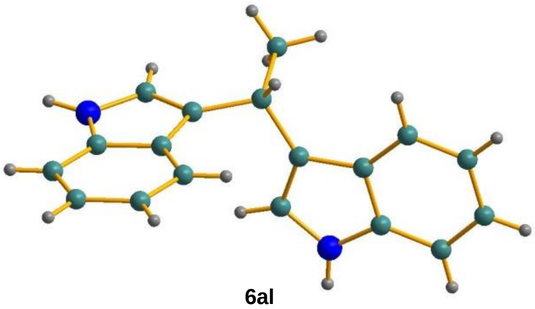

6al

Crystal data of **6al**: [$C_{18}H_{16}N_2$], orthorhombic, $P2_12_12_1$, a = 6.5030(13) Å, b = 9.927(2) Å, c = 21.129(4) Å, Z = 4, M_r = 260.33 g mol^{-1}, V = 1363.9(3) Å^3, D_{calcd} = 1.268 g cm^{-3}, λ(Mo Kα) = 0.71073 Å, T = 173 K, μ = 0.075 mm^{-1}, 7915 reflections collected, 2518 unique (R_{int} = 0.0438), $R1(F_o)$ = 0.0374 [$I > 2\sigma(I)$], $wR2$ (F_o^2) = 0.0692 (all data), GOF = 1.031. CCDC 1007025.

References

1. Peng, Y.-Y.; Zhang, Q.-L., Yuan, J.-J., Cheng, J.-P. *Chin. J. Chem.* **2008**, *26*, 2228-223.
2. Xu, H.-Y.; Zi, Y.; Xu, X.-P.; Wang, S.-Y.; Ji, S.-J. *Tetrahedron* **2013**, 69, 1600-1605.
3. Ramesh, C.; Banerjee, J.; Pal, R.; Das, B. *Adv. Synth. Catal.* **2003**, *345*, 557-559.
4. An, L.-T.; Ding, F.-Q., Zhou, J.-P.; Lu, X.-H., Zhang, L.-L. *Chin. J. Chem.* **2007**, *25*, 822-827.
5. Ji, S.-J.; Zhou, M.-F.; Gu, D.-G.; Jiang, Z.-Q.; Loh, T.-P. *Eur. J. Org. Chem.* **2004**, 1584-1587.
6. Khalafi-Nezhad, A.; Parhami, A.; Zare, A.; Moosavi Zare, A. R.; Hasaninejad, A.; Panahi, F. *Synthesis* **2008**, 617–621.
7. Veluri, R.; Oka, I.; Wagner-Döbler, I.; Laatsch, H. *J. Nat. Prod.* **2003**, *66*, 1520–1523.
8. Magesh, C. J.; Nagarajan, R.; Karthik, M.; Perumal, P. T. *Appl. Catal., A: Gen.* **2004**, *266*, 1–10.
9. Thirupathi, P.; Kim, S. S. *J. Org. Chem.* **2010**, *75*, 5240–5249.
10. Dhiman, S.; Ramasastry, S. S. V. *Org. Biomol. Chem.* **2013**, *11*, 8030–8035.
11. Pal, R.; Khannobis, S.; Sarkar, T. *Chem. J.* **2013**, *3*, 7–12.
12. Ramachandiran, K.; Muralidharan, D.; Perumal, P. T. *Tetrahedron Lett.* **2011**, *52*, 3579–3583.
13. CrysAlisPro, Agilent Technologies, Version 1.171.35.11. Multi-scans absorption correction with SCALE3 ABSPACK scaling algorithm.
14. G. M. Sheldrick, SHELXL-97, Program for Crystal Structure Refinement, University of Göttingen, Göttingen,Germany, 1997.
